# Gut microbiota in pregnant Malaysian women: a comparison between trimesters, body mass index and gestational diabetes status

**DOI:** 10.1186/s12884-022-04472-x

**Published:** 2022-02-24

**Authors:** Bahiyah Abdullah, Suzanna Daud, Mohd Shafiq Aazmi, Mohd Yusri Idorus, Mas Irfan Jaya Mahamooth

**Affiliations:** 1 Maternofetal and Embryo (MatE) Research Group, Faculty of Medicine, Universiti Teknologi MARA (UiTM), Sungai Buloh Campus, Sungai Buloh, Selangor Malaysia; 2 Department of Obstetrics and Gynaecology, Faculty of Medicine, Universiti Teknologi MARA (UiTM), Sungai Buloh Campus, Sungai Buloh, Selangor Malaysia; 3 School of Biology, Faculty of Applied Science, Universiti Teknologi MARA (UiTM), Shah Alam, Selangor Darul Ehsan Malaysia; 4 Faculty of Medicine, Institute of Medical Molecular Biotechnology, Universiti Teknologi MARA (UiTM), Sungai Buloh Campus, Sungai Buloh, Selangor Malaysia

**Keywords:** Gut microbiota, Pregnancy, Gestational diabetes mellitus, Underweight, Obese

## Abstract

**Background:**

The primary purpose of the study is to determine the variation of gut microbiota composition between first (T1) and third trimester (T3); gestational diabetes mellitus (GDM) and non-gestational diabetes mellitus (NGDM); and also within a different category of Body Mass Index (BMI) of selected pregnant Malaysian women.

**Methods:**

A prospective observational study on selected 38 pregnant Malaysian women attending a tertiary medical centre was carried out. Those with preexisting diabetes, metabolic syndrome or any other endocrine disorders were excluded. GDM was determined using oral glucose tolerance test (OGTT) while BMI was stratified as underweight, normal, pre-obese and obese. Fecal samples were then collected during the first trimester (T1) and the third trimester (T3). The V3-V4 region of 16S rRNA gene amplicon libraries were sequenced and analyzed using QIIME (version 1.9.1) and METAGENassist.

**Results:**

Twelve women (31.6%) were diagnosed as GDM. A trend of lower α-diversity indices in GDM, pre-obese and obese pregnant women were observed. Partial Least Squares Discriminant Analysis (PLS-DA) shows a clustering of gut microbiota according to GDM status and BMI, but not by trimester. Genera *Acidaminococcus, Clostridium, Megasphaera* and *Allisonella* were higher, and *Barnesiella* and *Blautia* were lower in GDM group (*P* < 0.005). Obese patients had gut microbiota that was enriched with bacteria of Negativicutes and Proteobacteria class such as *Megamonas, Succinatimonas* and *Dialister* (*P* < 0.005)*.* The normal and mild underweight profiles on the other hand had a higher bacteria from the class of Clostridia (*Papillibacter, Oscillibacter, Oscillospira, Blautia, Dorea)* and Bacteroidia (*Alistipes, Prevotella, Paraprevotella)* (*P* < 0.005)*.*

**Conclusion:**

The prevalence and variation of several key bacteria from classes of Negativicutes, Clostridia and Proteobacteria has potential metabolic links with GDM and body weight during pregnancy which require further functional validation.

**Supplementary Information:**

The online version contains supplementary material available at 10.1186/s12884-022-04472-x.

## Background

Pregnancy is a fascinating biological process that involves simultaneous changes physiologically, some of which have well been established, such as metabolic and hormonal alterations. However, only in the last decade has the importance of the gut microbiota in pregnancy been recognized [1].

There are millions of bacteria present in the gut, the majority of which are commensals. Although the actual composition of the gut microbiota is unclear, current research has revealed that 80–90% of bacteria morphologies belong to two phyla: Bacteroides and Firmicutes [2]. In addition to nutritional consumption, antibiotics, stress, and obesity; pregnancy has been proven to cause alteration in the gut microbiota composition.

The alteration in gut microbiota composition in pregnancy is accompanied by weight gain, insulin insensitivity, and increased cytokines that suggest inflammation. All of these changes are similar to those reported in people with metabolic syndrome [3]. These modifications were thought to be essential to accommodate the normal pregnancy demand.

To date, there is still a lack of information on gut microbiota profile among the pregnant Malaysian women population. With the rising trend of obesity among women of reproductive age, it is crucial to understand the composition of the gut microbiota as they are at risk of developing gestational diabetes later.

Knowledge on gut microbiota composition will allow it to be used as a platform to explore the role of modulation of the gut microbiota as a preventive and therapeutic tool in the treatment of gestational diabetes. Even though gut microbiota pattern has been reported in other countries, contrasting ethnic, cultural and dietary practices have been associated with different gut microbiota profile [4]. Hence, this study is crucial to observe whether there is any discrepancy with the published findings.

Therefore, this study aimed to determine the gut microbiota composition in the T1 and T3 among pregnant Malaysian women, to demonstrate its composition between women with gestational diabetes mellitus (GDM) and non-GDM (NGDM) and in different BMI categories.

## Methods

### Study design

This was a prospective observational study involving 38 women in a tertiary medical centre in Malaysia. All pregnant women who attended the antenatal clinic as outpatient that met the inclusion criteria were offered to participate. The inclusion criterias were: (i) Pregnant patients in the first trimester (T1); (ii) Malaysian; (iii) Be willing to be followed up until the third trimester (T3) and (iv) Agreeable to undergo Oral Glucose Tolerance Test (OGTT). The exclusion criteria were: (i) any known case of preexisting diabetes mellitus, metabolic syndrome or any other endocrine disorders and (ii) on any antibiotics /prebiotics/ probiotics during or in the past four weeks prior to recruitment.

The first trimester was defined as any pregnancy less than 13 weeks of gestation and the third trimester was any pregnancy beyond 27 weeks of gestation. GDM was diagnosed based on the OGTT result, a diagnostic test for GDM recommended by the national guideline. It was performed in the antenatal clinic using 75 g oral glucose. A fasting blood sample will be taken, followed by another blood sample taken two hours after consuming the oral glucose drink prepared (which they need to complete it within five minutes). If either fasting blood glucose is more than 5.1 mmol/L or 2-h post-prandial glucose is more than 7.8 mmol/L, they were diagnosed as GDM. Others were classified as non-GDM (NGDM).

### Sample size calculation

The sample size is determined based on the study by Collado et. al. (2008), who found the Bacteroides-Prevotella group count in fecal samples at first trimester was 9.74 (9.62, 9.87) log fecal cells/g and the Bacteroides-Prevotella group count in fecal samples at third trimester was 10.36 (10.27,10.45) log fecal cells/g. By taking α = 0.05, 80% power of the study, the standard deviation for T1 was 0.12, the standard deviation for T3 was 0.09, and estimated mean difference of 0.62, the sample size required for this study is 28 using the following formula:

$$\begin{array}{l}\mathrm{n}\hspace{0.17em}=\hspace{0.17em}{({\mathrm{Z}}_{\mathrm{\alpha }}\hspace{0.17em}+\hspace{0.17em}{\mathrm{Z}}_{\upbeta })}^{2} \frac{{\left({\upsigma }_{1}+{\upsigma }_{2}\right)}^{2}}{\mathrm{d}}\\ {(9.74\hspace{0.17em}+\hspace{0.17em}10.36)}^{2} \frac{{\left(0.12+0.09\right)}^{2}}{0.62}\\ \begin{array}{l}=\hspace{0.17em}404.01 (0.0711)\\ \hspace{0.17em}=\hspace{0.17em}28\end{array}\end{array}$$

By adjusting the 10% attrition rate, the minimum sample size in this study is 31.

### Data collection

The participants were asked to fill in a study proforma enquiring the participants’ basic demographic details. Anthropometric measurements were taken by trained nursing staff. Body Mass Index (BMI) was calculated and participants were categorised based on the World Health Organisation recommendation; underweight (below 18.5 kg/m^2^), normal (18.5–24.9 kg/m^2^), pre-obese (25.0–29.9 kg/m^2^) and obese (30 kg/m^2^ and above). The participants were then asked to give their stool samples during the first trimester. Sample collection, preservation and storage were performed using Stool Nucleic Acid Collection and Preservation Tube (NORGEN, Canada). A total of 2 g samples were collected and filled into the collection tubes, gently mixed until the stool is well submerged under the liquid preservative. They were required to have an OGTT test at least once during this pregnancy. Once they reach the third trimester, they were again asked to give another fecal sample using the same kit. Patients were followed up until delivery, and delivery details was be obtained including the mode of delivery and the baby’s anthropometric measurement.

### DNA extraction

Total DNA of the stool samples was extracted from approximately 400 µl of liquid samples by Stool DNA Isolation Kit (NORGEN, Canada) following the manufacturer's instruction. The final DNA concentration and purity were determined by SpectraMax QuickDrop Micro-Volume Spectrophotometer (Molecular Devices, USA). The ratio of sample absorbance at 260 and 280 nm was used to assess the purity of the DNA. The DNA integrity was assessed by running a 1% agarose gel electrophoresis (Sigma-Aldrich, USA) and stained with SYBR Safe DNA Gel Stain (Invitrogen, USA). Extracted DNA was stored at -20˚C pending sequencing analysis.

### 16S ribosomal ribonucleic acid metagenome analysis

The V3-V4 hypervariable regions of the bacteria 16S rRNA gene were amplified with a set of primers 338F (5′-ACTCCTACGGGAGGCAGCA-3′) and 806R (5′-GGACTACHVGGGTWTCTAAT-3′) by thermocycler PCR system (27 cycles for each sample) (GeneAmp 9700, ABI, USA) according to the standard protocols by Majorbio Bio-Pharm Technology Co. Ltd. (Shanghai, China). The PCR reactions were conducted using the following conditions: three minutes of denaturation at 95 °C, 27 cycles of 30 s at 95 °C, 30 s for annealing at 55 °C, and 45 s for elongation at 72 °C, and a final extension at 72 °C for 10 min.

PCR amplification was performed using TransStart Fastpfu DNA Polymerase (TransGen AP221-02) under 20 μl reaction containing 4 μL of 5 × FastPfu Buffer, 2 μL of 2.5 mM dNTPs, 0.8 μL of each primer (5 μM), 0.4 μL of FastPfu Polymerase, 0.2 μL BSA and 10 ng of template DNA. The PCR products were detected by gel electrophoresis in 2% agarose gel. Amplicons were extracted from the agarose gels and purified using the AxyPrep DNA Gel Extraction Kit (AxygenBiosciences, USA) and quantified using QuantiFluor™-ST (Promega, USA) following the manufacturer's protocol.

The sample libraries were pooled in equimolar and paired-end sequenced (2 × 300) on an Illumina MiSeq platform (Illumina, USA) according to the standard protocols by Majorbio Bio-Pharm Technology Co. Ltd. (Shanghai, China). Assembly, binning, and annotation of DNA sequences were performed. Raw fastq files were demultiplexed, quality-filtered using QIIME (version 1.9.1) with the following criteria: The 300 bp reads were truncated at any site receiving an average quality score < 20 over a 50 bp sliding window, discarding the truncated reads that were shorter than 50 bp, exact barcode matching, two nucleotide mismatch in primer matching, reads containing ambiguous characters were removed and only sequences that overlap longer than 10 bp were assembled according to their overlap sequence. Reads which could not be assembled were discarded. The taxonomy of each 16S rRNA gene sequence was analyzed by RDP Classifier (http://rdp.cme.msu.edu/) against the Silva (SSU123) 16S rRNA database using a confidence threshold of 0.7. OTU-level species accumulation curve was used to assess the sequencing depth and species richness from the result of sampling. Alpha diversity indices, including Chao 1 richness, Abundance-based Coverage Estimator (ACE) metric, Shannon-Weiver curve and Simpson Index were calculated using Mothur.

### Statistical and comparative metagenomics analysis

Clinical baseline characteristics are presented as mean ± standard deviation. Spearman's rank correlation coefficient analysis was carried out. All statistical analyses were carried out using SPSS version 22 (IBM Corp., Armonk NY, USA). Statistical significance was defined as a *P*‐value < 0.05. Comparative metagenomics analysis of the 16S rRNA gut microbiota profiles were performed between different gestational trimesters (T1 vs T3), GDM status (GDM vs NGDM), BMI (Normal vs Abnormal) and also among the BMI subgroups (Underweight, Normal, Pre-obesity, Obesity) using METAGENassist [5].

Row-wise normalization by sum was performed on the bacterial relative abundance data matrix to normalize the inherent differences within metagenomes (sequencing depth). Column-wise normalization by log_10_ transformation was employed to obtain a more normal/Gaussian distribution of each bacterial taxa before statistical analysis is performed. Univariate statistics such as Student T-test and Anova with Post hoc Fisher’s LSD test with the significant *P* value less than 0.05 were used to determine any significant differences in the abundance of each phylum and genera between trimesters.

Multivariate analysis using the supervised model PLSDA of *β*-diversity was used to reveal any similarity or clustering pattern in the community structure between the gestational trimester, GDM status and BMI groups. The performance of the discriminant pattern from the PLSDA model was evaluated based on R^2^ values (less than 0.33, weak; 0.33–0.67, moderate; 0.67 and above, substantial model [6]. Loading plots from PLSDA and variable importance in projection (VIP) were used to determine the importance of each phylum and genus in each community profile.

## Result

### Description of the study cohort

Thirty-eight Malaysian women were recruited, and their clinical characteristics were presented in Table 1. In this study, 12 pregnant women (*n* = 12/38, 31.6%) were diagnosed with GDM. There were no significant differences in other clinical characteristics between the GDM and NGDM groups of participants. The majority of the recruited pregnant women had normal BMI (*n* = 16/38, 42.1%). There were 13 pre-obese (34.2%), followed by seven obese (18.2%) and two underweight patients (both are under the category of mild thinness) (5.3%) were also recorded.

**Table 1:** Clinical characteristics of subjects

	**ALL**	**GDM**	**NGDM**	***p*—value**
N	38	12	26	
Age				
mean (sd)	30.55(4.03)	30.42 (3.801)	30.62 (4.205)	0.890
Parity				
median(range)	1 (0–5)			
Educational status, n(%)				
Secondary	5(13.2)	2(16.7)	3(11.5)	0.643
Tertiary	33(86.8)	10(83.3)	23(88.5)	
Occupation, n(%)				0.021
Unemployed / Housewife	6(15.8)	4(33.3)	2(7.7)	
Non-professional	14(36.8)	1(8.3)	13(50.0)	
Professional	18(47.4)	7(58.3)	11(42.3)	
Monthly income, mean(sd)				
B40	11(28.9)	4(33.3)	7(26.9)	0.899
M40	14(36.8)	6(50.0)	15(57.7)	
T20	6(15.8)	2(16.7)	4(15.4)	
MGTT (Fasting), mean(sd)	4.49(0.55)	5.01 (0.46)	4.25 (0.39)	< 0.001*
MGTT (2HPP), mean (sd)	6.59(1.75)	8.64 (1.31)	5.66 (0.92)	< 0.001*
BMI (kg/m^2^), mean(sd)	25.34 (5.54)	27.37 (4.61)	24.41 (5.75)	0.126
Booking Systolic BP (mmHg), mean(sd)	114.03 (12.07)	117.45 (11.77)	112.32 (12.12)	0.256
Booking Diastolic BP (mmHg) mean(sd)	70 .06 (8.81)	73.36 (8.52)	68.41 (8.67)	0.130
Booking heart rate (beats/min), mean(sd)	86.80 (10.97)	91.00 (8.12))	84.37 (11.85)	0.112

*T1* First trimester of pregnancy, *T3* Third trimester of pregnancy, *GDM* Gestational diabetes mellitus, *NGDM* Non-Gestational diabetes mellitus, *BMI* Body mass index, *BP* Blood pressure, *MGTT* Modified Glucose Tolerance Test

^*^Statistically significant at P < *0.05*

### The biodiversity of the gut microbiota

The bacterial biodiversity in the gut microbiota of the pregnant women in T1 and T3 were analyzed according to pregnancy trimester (Table 2), GDM status (Table 3) and BMI groups (Table 4). The mean number of reads for the above-mentioned grouping were ranging from 303,934 to 51,792 reads. The mean number of operational taxonomic units (OTUs) across the groups ranged from 481 to 1051 OTUs. Coverage indexes for all the groups were more than 99%, indicating that the sequences of all the gut microbiota in each sample were detected.

**Table 2:** Bacterial biodiversity in gut microbiota in the study cohort

	**T1**	**T3**
N	38	38
Number of reads	50,989 ± 10,447	47,415 ± 12,327
Number of OTUs	908 ± 397	796 ± 415
Coverage	0.9954 ± 0.0015	0.9955 ± 0.0016
Ace	1127 ± 454	1013 ± 462
Chao	1138 ± 470	1014 ± 483
Shannon	4.14 ± 0.65	3.89 ± 0.77
Simpson	0.07 ± 0.05	0.09 ± 0.08

Data presented as Mean ± SD

*T1*, First trimester of pregnancy, *T3* Third trimester of pregnancy

No significant differences (*P* > 0.05) were observed for all the diversity indices

**Table 3:** Bacterial biodiversity in gut microbiota between GDM and NGDM cohort

	**GDM**		**NGDM**	
	**T1**	**T3**	**T1**	**T3**
N	12	12	26	26
Number of reads	49,812 ± 9762	48,538 ± 15,631	51,532 ± 10,891	46,896 ± 10,793
Number of OTUs	672 ± 232	849 ± 455	1017 ± 414	771 ± 403
Coverage	0.9963 ± 0.0010	0.9951 ± 0.0021	0.9950 ± 0.0015	0.9957 ± 0.0014
Ace	868 ± 289	1093 ± 511	1247 ± 471	977 ± 443
Chao	863 ± 301	1100 ± 535	1265 ± 484	975 ± 463
Shannon	3.73 ± 0.61	3.80 ± 0.82	4.32 ± 0.60	3.92 ± 0.75
Simpson	0.0914 ± 0.0699	0.0948 ± 0.0665	0.0579 ± 0.0433	0.0887 ± 0.0854

Data presented as Mean ± SD

*SD* Standard Deviation, *T1* First trimester of pregnancy, *T3* Third trimester of pregnancy

**Table 4:** Bacterial biodiversity in gut microbiota between BMI groups

	**Underweight**		**Normal BMI**		**Pre-obese**		**Obese**	
	**T1**	**T3**	**T1**	**T3**	**T1**	**T3**	**T1**	**T3**
N	2	2	16	16	13	13	7	7
Number of reads	303,934 ± 345	49,716 ± 11,930	52,909 ± 8622	40,529 ± 8803	51,409 ± 10,380	43,285 ± 13485	51,549 ± 11,269	51,792 ± 10,740
Number of OTUs	640 ± 28	484 ± 77	1051 ± 62	896 ± 505	866 ± 361	711 ± 303	734 ± 246	813 ± 408
Coverage	0.9938 ± 0.00003	0.9962 ± 0.0017	0.9952 ± 0.0015	0.9955 ± 0.0018	0.9957 ± 0.0014	0.9953 ± 0.0016	0.9961 ± 0.0013	0.9959 ± 0.0016
Ace	838.5 ± 40	707 ± 189	1280 ± 532	1107 ± 568	1077 ± 419	923 ± 309	953 ± 291	1055 ± 487
Chao	836.5 ± 45	642 ± 95	1293 ± 546	1118 ± 586	1095 ± 435	919 ± 345	952 ± 314	1060 ± 496
Shannon	4.37 ± 0.25	3.09 ± 0.94	4.28 ± 0.65	4.05 ± 0.84	4.11 ± 0.64	3.87 ± 0.66	3.78 ± 0.72	3.76 ± 0.72
Simpson	0.03 ± 0.0096	0.2213 ± 0.2377	0.06 ± 0.0410	0.0731 ± 0.0574	0.0676 ± 0.05	0.0922 ± 0.0704	0.1005 ± 0.0849	0.0901 ± 0.0670

Data presented as Mean ± SD

*SD* Standard Deviation *T1* First trimester of pregnancy *T3* Third trimester of pregnancy

The species accumulation assessment using the Shannon rarefaction curve showed a plateau and saturation phase, indicating sufficient sequencing depth, and the sample size was sufficient to capture the overall richness of gut microbiota composition in this study [see Additional file 1]. At a rarefied sequencing depth of 23,433 reads, the mean number of the observed OTUs and diversity indices (Ace, Chao, Shannon and Simpson) between trimester of pregnancy, GDM and NGDM as well as between the BMI groups were not significantly different. However, a trend of relatively lower diversity indices (Ace, Chao, Shannon and Simpson) were observed in the gut microbiota profiles of GDM than in NGDM pregnant women. Similarly, there was a trend of lower diversity indices in pre-obese and obese pregnant women than women with normal BMI.

### Gut microbiota profile in first and third trimester of pregnancy

**Fig. 1:**
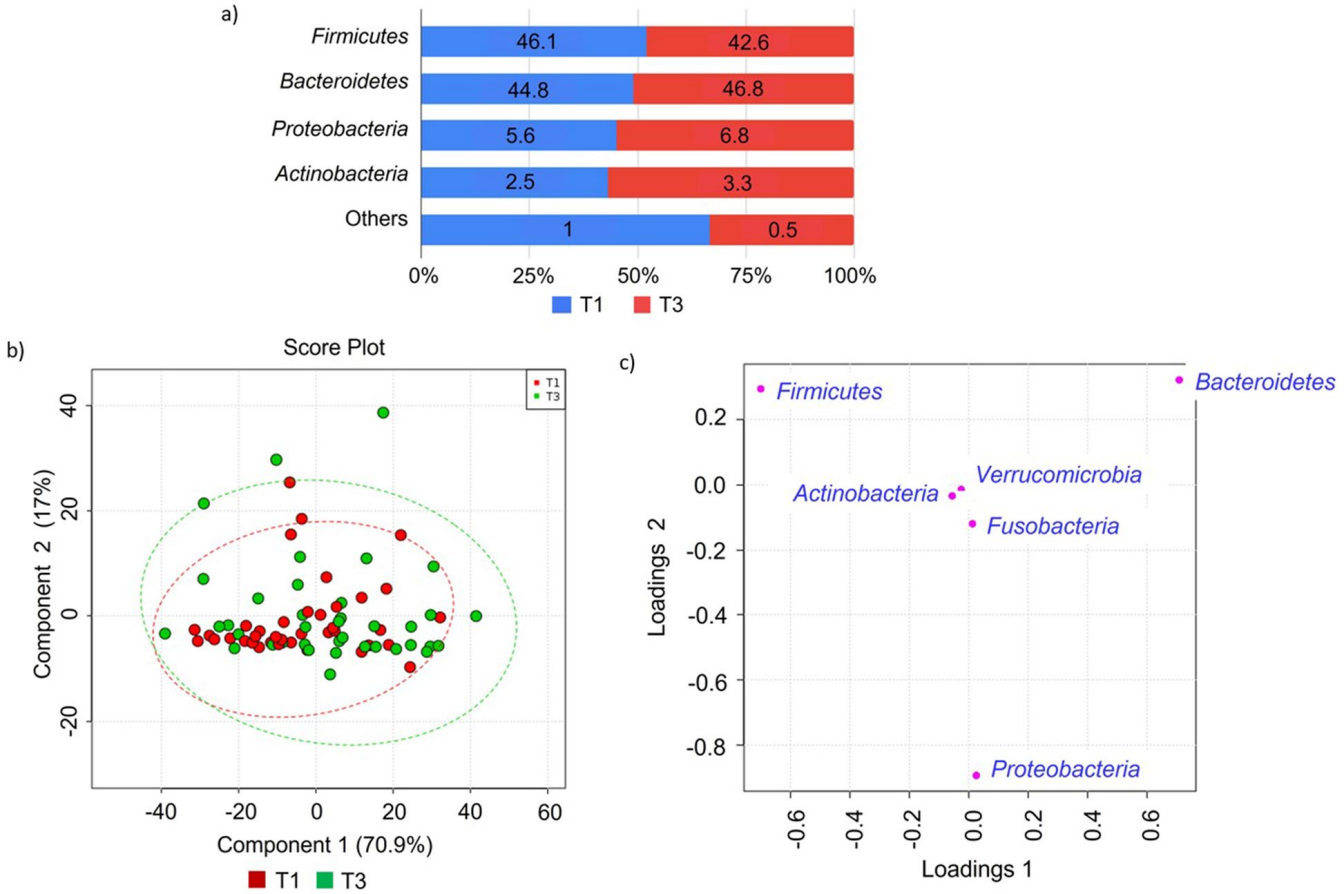
Community structure in women during the first (T1) and third trimester of pregnancy (T3) at taxonomic phyla level. **a** Relative abundance of bacterial phyla at T1 (*N* = 38) and T3 (*N* = 38). Data represented as mean relative abundance of the phylum. **b** PLSDA score plot shows a similar community structure in T1 (red dots) and T3 (green dots). The line ellipses on the PLSDA score plot indicate the 95% confidence interval. **c** Loading plot shows three dominant phyla (Firmicutes, Bacteroidetes and Proteobacteria) in the T1 and T3 profile of gut microbiota

Phyla Firmicutes, Bacteroidetes, Proteobacteria and Actinobacteria represented 99% and 99.5% of gut microbiota composition in T1 and T3 (Fig. 1). In T1, Firmicutes was the dominant phyla (46.1%) and the trend shifted in T3 in which Bacteroidetes was prevalent (46.8%) (Fig. 1a). We found no statistically significant differences between the relative abundances of phyla between pregnancy trimesters. No clustering pattern of gut microbiota profile according to the trimester of pregnancy (T1 and T3) based on the PLSDA analysis was also observed (Fig. 1b). However, the PCA loading plot shows that Firmicutes, Bacteroidetes and Proteobacteria were the key phyla in the trend mentioned above (Fig. 1c).

**Fig. 2:**
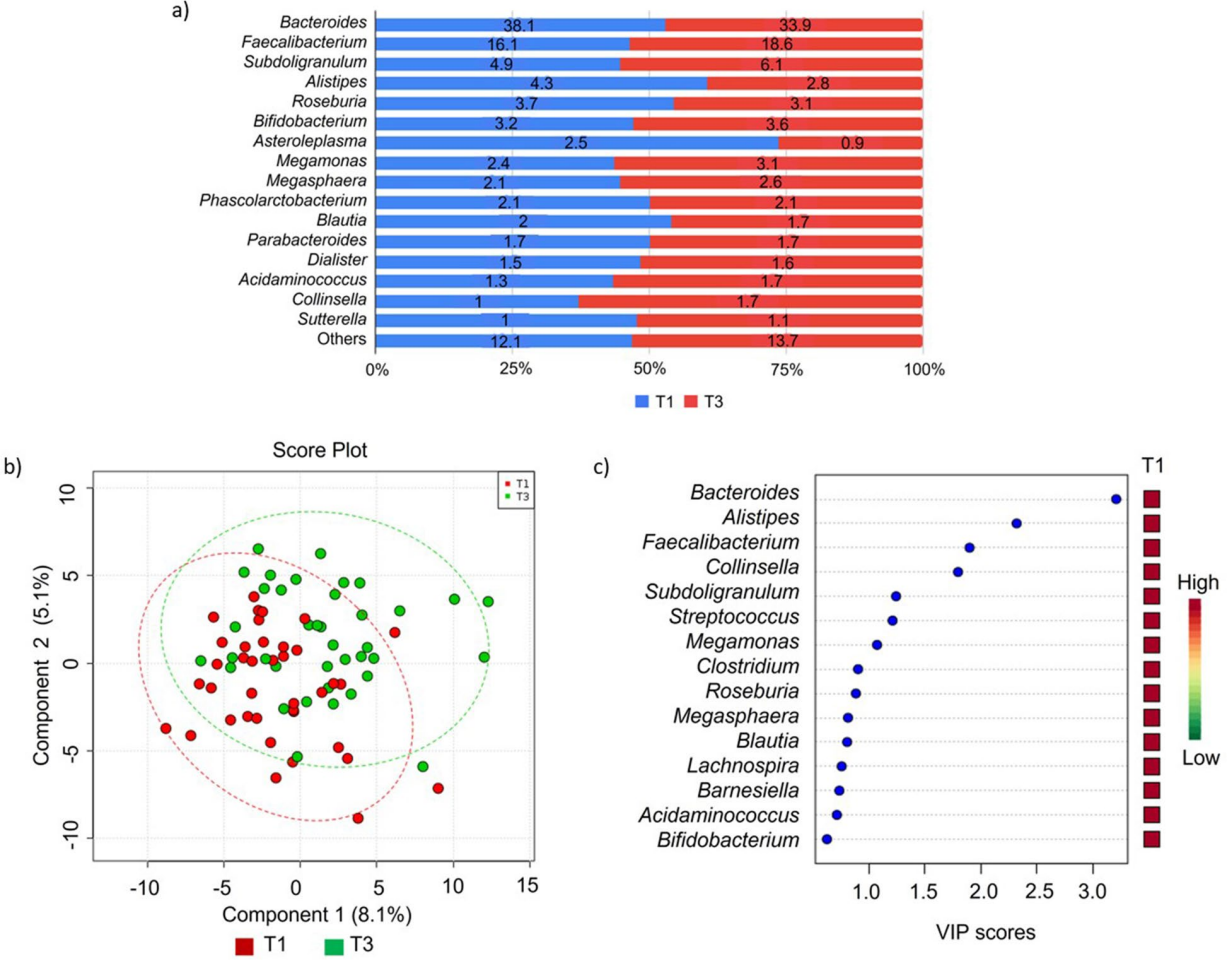
The community structure of gut bacterial genera in the first (T1) and third trimester of pregnancy (T3). **a** Relative abundance of bacteria genera at T1 (*N* = 38) and T3 (*N* = 38). Data represented as the mean relative abundance. **b** PLSDA score plot shows a similar structure between T1 (red dots) and T3 (green dots). The line ellipses on the PLSDA score plot indicate the 95% confidence interval. **c** Four key bacterial genera in T1 and T3 profiles with VIP score more than 1.5 were identified (*Bacteroides, Alistipes, Faecalibacterium, Collinsella*)

The prevalent bacterial genus was presented in Fig. 2. Genus *Bacteroides* and *Faecalibacterium* were the most prevalent genera representing more than 50% of the gut microbiota in T1 and T3 profiles (Fig. 2a). There is no clustering pattern observed from the PLSDA score plots (Fig. 2b). However, *Bacteroides, Alistipes, Faecalibacterium* and *Collinsella* were identified as the dominant bacterial genus in both community structures of gut microbiota in T1 and T3 with a VIP score of more than 1.5 (Fig. 2c). However, the differences in the relative abundance of each genus between T1 and T3 were not statistically significant by Student T-test.

### Gut microbiota profile in GDM versus NGDM patients

**Fig. 3:**
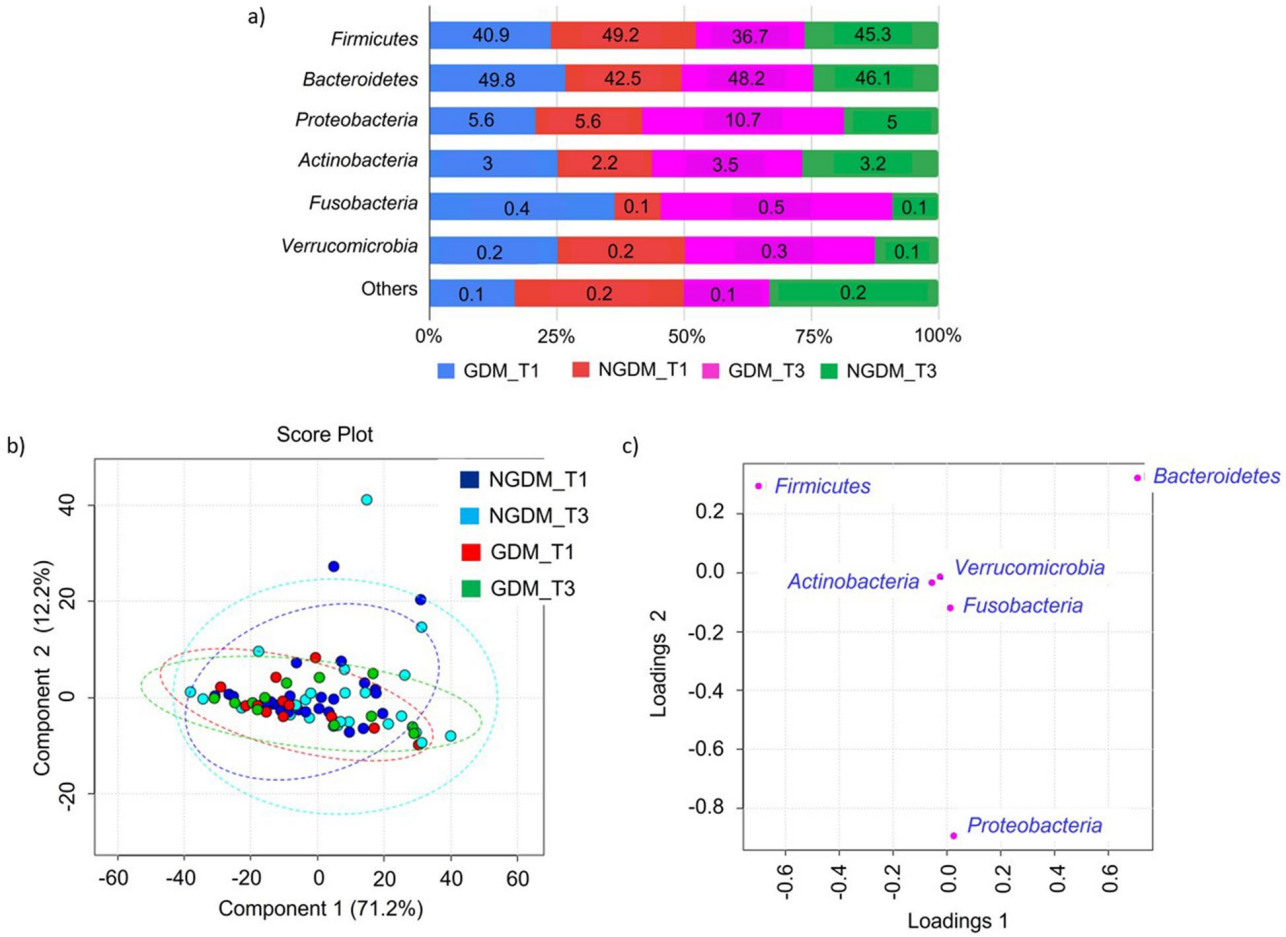
Community structure in women with and without gestational diabetes. **a** Relative abundance of bacterial phyla in GDM (*N* = 12) and NGDM (*N* = 26) during T1 and T3 pregnancy. Data represented as mean relative abundance. **b** PLSDA score plot shows a similar community structure between GDM T1 (red dots), GDM T2 (green dots), NGDM T1 (blue dots) and NGDM T3 (cyan dots). The line ellipses on the PLSDA score plot indicate the 95% confidence interval. **c** Loading plots show three dominant phyla in a community (Firmicutes, Bacteroidetes and Proteobacteria). GDM, Gestational diabetes mellitus; NGDM, Non-Gestational diabetes mellitus; T1, First trimester of pregnancy; T3, Third trimester of pregnancy

A majority (> 99%) of the identified gut microbiota in both GDM and NGDM patients during T1 and T3 are from the phyla Firmicutes, Bacteroidetes, Proteobacteria, Actinobacteria and Fusobacteria (Fig. 3). We noted that *Bacteroidetes* was the dominant phyla in the GDM group throughout their pregnancy T1 and T3. The Firmicutes were seen prevalent in T1 and T3 of the NGDM group. Proteobacteria was seen to have increased almost two folds in the GDM group during T3 (10.7%) as compared to in T1 (5.6%) (Fig. 3a). Any clustering pattern of gut microbiota according to the gestational diabetic status and pregnancy trimesters was also not observed from the PLSDA analysis (Fig. 3b). Trends observed on the relative abundances of Firmicutes, Bacteroidetes and Proteobacteria were also reflected in the loading plot of PCA indicating the key phyla (Fig. 3c). However, no statistically significant differences were observed in the relative abundances between groups and pregnancy trimesters using ANOVA.

**Fig. 4:**
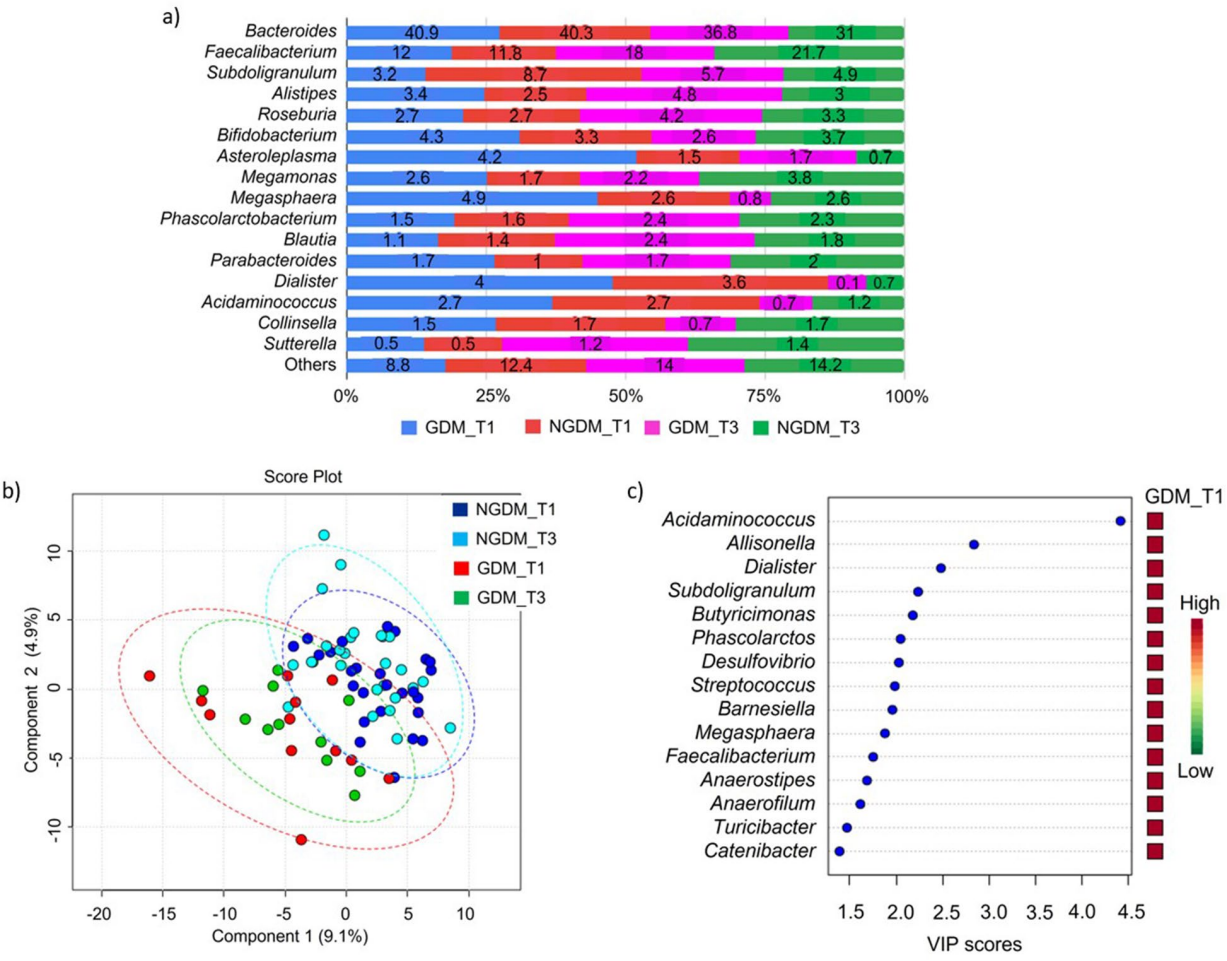
Community structure of bacterial genera in women with and without gestational diabetes. **a** Relative abundance of bacterial genera in GDM (*N* = 12) and NGDM (*N* = 26) during T1 and T3 pregnancy. Data represented as mean relative abundance **b** PLSDA score plot shows a discriminant pattern for differentiating community structure between GDM T1 (red dots), GDM T2 (green dots), NGDM T1 (blue dots) and NGDM T3 (cyan dots). The ellipses line is the confidence interval of 95%. **c** The key bacterial genera in the GDM-associated gut microbiota with VIP score more than 1.5. GDM (Gestational diabetes mellitus), NGDM (Non-Gestational diabetes mellitus), T1 (First trimester of pregnancy), T3 (Third trimester of pregnancy), VIP (Variable importance project)

At the genus level, *Bacteroides* and *Faecalibacterium* were the dominant genera representing more than 50% of the gut microbiota community structure in GDM and NGDM groups (Fig. 4a). A discriminant pattern was observed between GDM-associated and NGDM-associated gut microbiota (R^2^ = 0.59), but not by the trimester of pregnancy (T1 and T3) (Fig. 4b). From the PLSDA model, 15 key genera with the highest VIP score (> 1.5) were identified contributing to the observed discriminant pattern of gut microbiota associated with gestational diabetes as shown in Fig. 4c. The genera were *Acidaminococcus, Allisonella, Dialister, Suddoligranulum, Butyricimonas, Phascolarctobacteria, Desulfovibrio, Streptococcus, Barnesiella, Megasphaera, Faecalibacterium, Anaerostipes, Anaerofiulum, Turicibacter and Catenibacter* (Fig. 4c).

Among the significant 85 genera in the PLSDA-VIP score list [see Additional file 2], six genera had statistically significant differences in relative abundances in at least one of the groups (GDM T1, NGDM T1, GDM T1, NGDM T3) after testing with ANOVA and Post hoc Fisher’s LSD (*P* < 0.05) (Table 5). The genera were *Acidaminococcus, Clostridium, Barnesiella, Blautia, Megasphaera,* and *Allisonella*. Among these, *Acidaminococcus, Clostridium, Megasphaera* and *Allisonella* were found significantly higher, and *Barnesiella* and *Blautia* were found significantly lower in women with GDM.

**Table 5:** Abundance of bacterial genera that differed between gestational diabetes and normal group in first and third trimester of pregnancy

Genus	*P* value	Fisher's LSD comparison
*Acidaminococcus*	0.027	GDM_T1 > NGDM_T1; GDM_T3 > NGDM_T1
*Clostridium*	0.034	GDM_T3 > GDM_T1; GDM_T3 > NGDM_T1; GDM_T3 > NGDM_T3
*Barnesiella*	0.034	NGDM_T1 > GDM_T1; NGDM_T1 > GDM_T3; NGDM_T3 > GDM_T3
*Blautia*	0.038	NGDM_T1 > GDM_T1; NGDM_T1 > GDM_T3
*Megasphaera*	0.045	GDM_T1 > NGDM_T1
*Allisonella*	0.046	GDM_T1 > NGDM_T1; GDM_T3 > NGDM_T1

> , higher in relative abundance (%), *GDM* Gestational diabetes mellitus, *NGDM* Non-Gestational diabetes mellitus, *T1* First trimester of pregnancy, *T3* Third trimester of pregnancy.

Significant *P* < 0.05 by ANOVA and Post hoc Fisher’s LSD

### Gut microbiota profile in normal and abnormal BMI groups

**Fig. 5:**
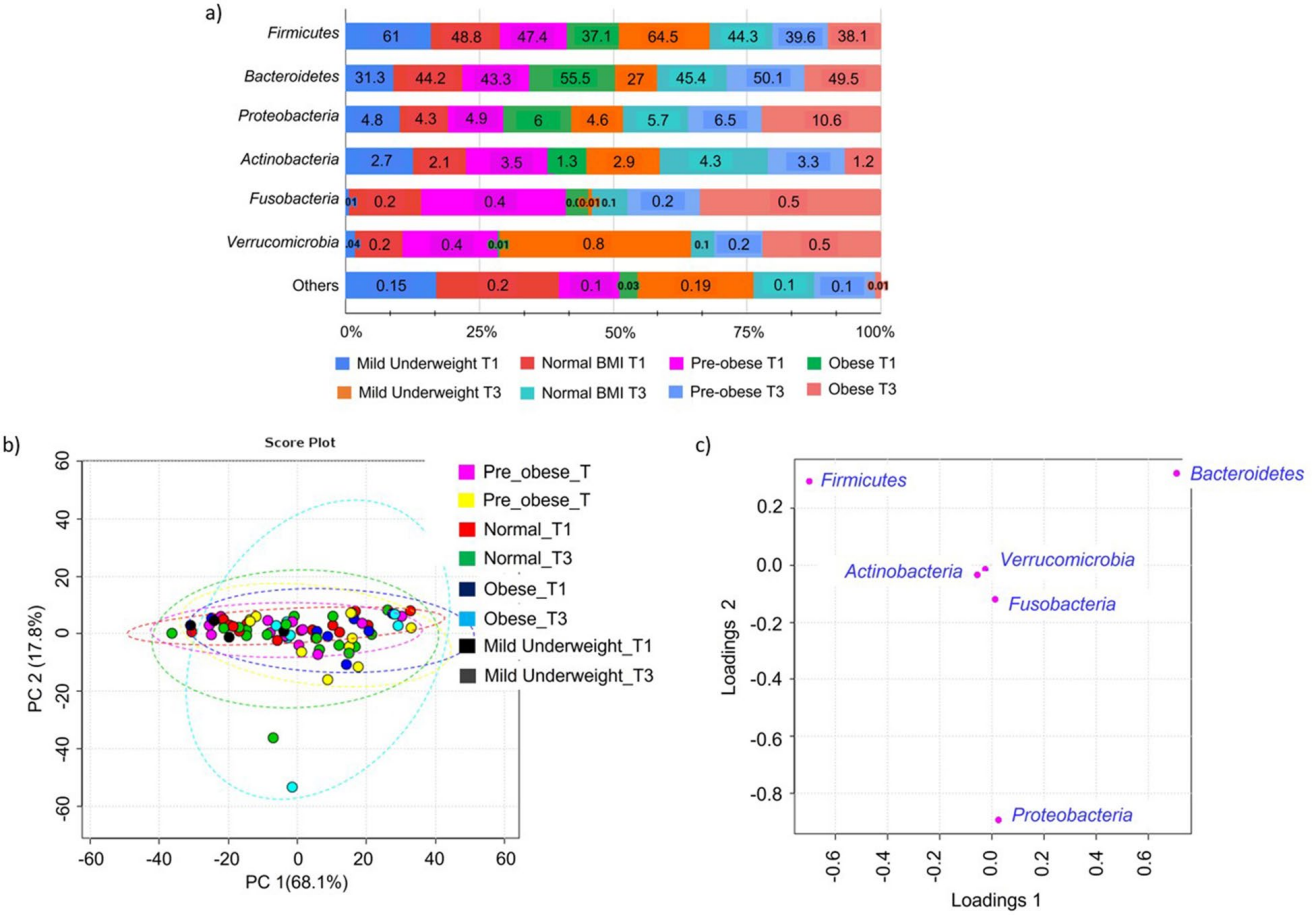
Community structure according to BMI in T1 and T3. **a** Relative abundance of bacterial phyla in Mild underweight (*N* = 2), Normal BMI (*N* = 16), Pre-obese (*N* = 13) and Obese (*N* = 7). **b** PCA score plots show a similar community structure across the BMI groups. **c** Loading plots indicate three dominant phyla in the profiled community. Data represented as mean relative abundance of the phylum. Body mass index, BMI; T1, First trimester of pregnancy; T3, Third trimester of pregnancy. The line ellipses on the PCA score plot indicate the 95% confidence interval

Four dominant phyla Firmicutes, Bacteroidetes, Proteobacteria and Actinobacteria represent the majority (> 99%) of the gut microbiota community across all the BMI groups (Fig. 5). The relative abundance of Firmicutes was seen highest in mild underweight patients (62.8%) followed by the normal BMI group (46.5%) as compared to pre-obese and obese groups. In contrast, Bacteroidetes were detected highest in obese (54.7%) and pre-obese (46.7%) groups (Fig. 5a). However, ANOVA testing shows no statistically significant differences in the relative abundances of each phylum between the BMI groups. The community structure of the microbiota at the phyla level across the BMI groups is similar as no clustering pattern was observed on the PCA score plot (Fig. 5b). Loading plots for the PCA support the finding on the dominant presence of Firmicutes, Bacteroidetes and Proteobacteria in the community within the BMI groups (Fig. 5c).

**Fig. 6:**
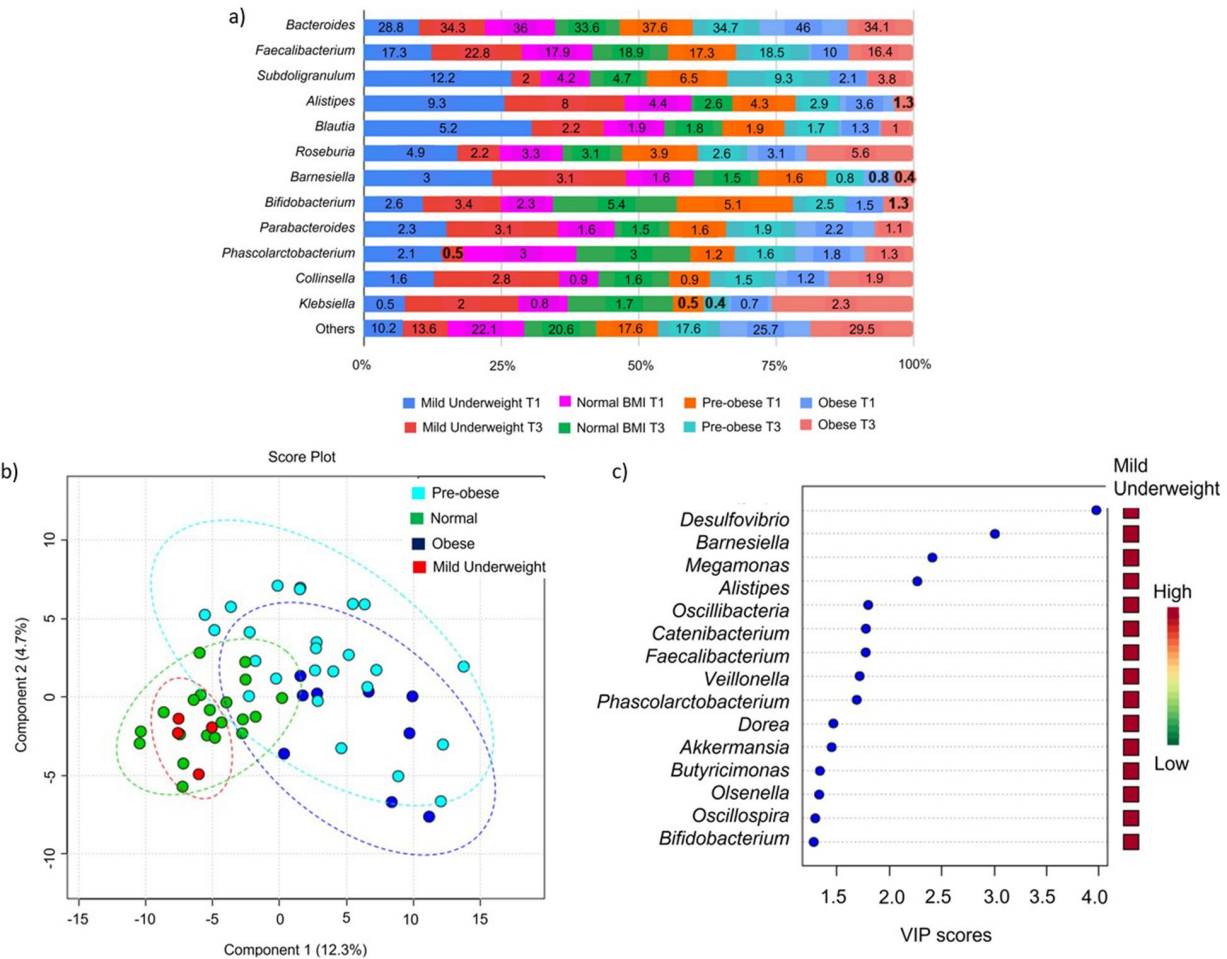
Community structure at the genera taxonomic level according to BMI in T1 and T3. **a** Relative abundance of bacterial genera in Mild underweight (*N* = 2), Normal BMI (*N* = 16), Pre-obese (*N* = 13) and Obese (*N* = 7). Data represented as mean relative abundance of the phylum. **b** PLSDA score plot shows a discriminant pattern between the community structure of Mild underweight (red dots), Normal BMI (green dots), Pre-obese (cyan dots) and Obese (blue dots) using combined datasets of T1 and T3 as one group. The ellipses line is the confidence interval of 95%. **c** The key bacterial genera in the BMI-associated gut microbiota with the highest VIP score (> 1.5). Body mass index, BMI; T1, First trimester of pregnancy; T3, Third trimester of pregnancy; VIP (Variable importance project)

Community structure at the genera taxonomic level according to BMI in T1 and T3 is further demonstrated in Fig. 6. A detailed inspection found that *Bacteroides* and *Faecalibacterium* are the dominant genera representing more than 50% of the identified gut microbiota across the BMI groups (Fig. 6a). Due to the limited number of variables from the mild underweight group (*N* = 2) which is not valid for tenfold cross-validation in the PLSDA model, we have to combine datasets of T1 and T3 in one group according to respective BMI grouping to fit the validation criteria. Besides that, the gut microbiota profile between T1 and T3 were found similar (Fig. 2b), thus we combined the data into one group.

The generated PLSDA score plot shows a discriminant pattern of gut microbiota between two overlapping clusters: (i) mild underweight and normal BMI, (ii) pre-obese and obese (R^2^ = 0.65) (Fig. 6b). There are 85 genera that were identified in contributing to the discriminant pattern observed on the PLSDA-VIP score list [see Additional file 3]. Out of this, 15 key genera in BMI-associated gut microbiota profiles with the highest VIP score (> 1.5) were observed. The genera were *Desulfovibrio, Barnesiella, Megamonas, Alistipes, Oscillibacter, Catenibacter, Faecalibacterium, Veillonella, Phascolarctobacteria, Dorea, Akkermansia, Butyricimonas, Olsenella, Ocsillospira* and *Bifidobacteria* (Fig. 6c)*.*

From Anova testing, there were 17 genera that had significant differences in relative abundance in at least one of the BMI grouping (*P* < 0.05) (Table 6). In the obese profile, *Megamonas, Succinatimonas* and *Dialister* were elevated whereas *Oscillibacter, Oscillospira, Butyricimonas, Alistipes, Prevotella* were reduced. Two genera which are *Barnesiella* and *Blautia* were found reduced in both obese and pre-obese profiles. In the normal body weight group, *Desulfovibrio* and *Dorea* were elevated. Within the mild underweight profile, we observed an elevation of *Porphyromonas, Papillibacter, Victivallis* and *Paraprevotella,* as well as a reduction in the relative abundance of *Megasphaera* (Table 6).

**Table 6:** Abundance of bacterial genera that differed between BMI groups

Genus	*P* value	Fisher's LSD comparison
*Porphyromonas*	0.002	Mild Underweight > Normal; Mild Underweight > Obese; Mild Underweight > Preobese
*Desulfovibrio*	0.009	Normal > Obese; Normal > Preobese
*Papillibacter*	0.005	Mild Underweight > Normal; Mild Underweight > Obese; Mild Underweight > Preobese
*Oscillibacter*	0.001	Mild Underweight > Obese; Normal > Obese; Normal > Preobese; Preobese > Obese
*Oscillospira*	0.002	Mild Underweight > Obese; Normal > Obese; Normal > Preobese; Preobese > Obese
*Megamonas*	0.003	Obese > Mild Underweight; Obese > Normal; Obese > Preobese
*Victivallis*	0.004	Mild Underweight > Normal; Mild Underweight > Obese; Mild Underweight > Preobese
*Barnesiella*	0.006	Mild Underweight > Obese; Mild Underweight > Preobese; Normal > Obese; Normal > Preobese
*Butyricimonas*	0.008	Mild Underweight > Obese; Normal > Obese; Preobese > Obese
*Blautia*	0.009	Mild Underweight > Obese; Mild Underweight > Preobese; Normal > Obese
*Dorea*	0.013	Normal > Obese; Normal > Preobese
*Alistipes*	0.014	Mild Underweight > Obese; Normal > Obese
*Prevotella*	0.022	Mild Underweight > Obese; Normal > Obese
*Megasphaera*	0.034	Normal > Mild Underweight; Obese > Mild Underweight; Preobese > Mild Underweight
*Paraprevotella*	0.039	Mild Underweight > Normal; Mild Underweight > Obese; Mild Underweight > Preobese
*Dialister*	0.041	Obese > Normal; Obese > Preobese
*Succinatimonas*	0.044	Obese > Preobese

> , higher in relative abundance (%), Significant *P* < 0.05 by ANOVA and Post hoc Fisher’s LSD

## Discussion

Pregnancy is often associated with an increase in bacterial load and dramatic changes in the taxonomic composition of the gut microbiota [3, 5, 6]. These substantial changes are manifested by decreased individual richness (α-diversity), increased inter-subject diversity (β-diversity), and shifts in the abundance of certain species [3].

The majority of the alterations were seen between the non-pregnant women or pregnant women in early pregnancy and those from advanced pregnancy. Hence, we believe our study comparing the pregnant women in T1 and T3 would add valuable findings in this area. However, in general, our findings indicate that pregnancy progression from T1 to T3 was not related to a substantial alteration in the variety and composition of pregnant women's gut microbiota biodiversity. The mean number of the observed OTUs and α-diversity (individual richness) indices (Ace, Chao, Shannon and Simpson) between the trimester of pregnancy was not significantly different. This contradicts the findings by Koren O. et al. (2012) that reported the presence of a significant reduction in within-subject diversity [3]. However, DiGiulio D. B. et. al. (2015) also found no significant trend in the Shannon diversity index of the gut microbiota composition during pregnancy [7].

The prevalence of GDM among our participants was 31.6%, similar to the prevalence of GDM in Malaysia which was reported to be 27.9% in 2017 [8]. Similarly, there was no statistically significant difference in gut microbiota α-diversity between pregnant women with GDM and NGDM, and between pregnant women with different BMI groups. However, a trend of relatively lower α-diversity indices (Ace, Chao, Shannon and Simpson) was observed in the gut microbiota profiles of GDM than in NGDM pregnant women. Likewise, there was a trend of lower α-diversity indices in pre-obese and obese pregnant women than women with normal BMI.

Our study also demonstrates that there was no statistically significant difference between the abundances of gut microbiota phyla and genera between T1 and T3. Similar to previously reported in the literature, the most dominant phyla were *Firmicutes*, *Bacteroidetes, Proteobacteria* and *Actinobacteria* [3] *Actinobacteria* and *Proteobacteria* both shows an increasing trend, and *Faecalibacterium* shows a decreasing trend, despite it was not statistically significant. Whereas *Bacteroides, Alistipes, Faecalibacterium* and *Collinsella* were identified as the dominant bacterial genus in both community structures of gut microbiota in T1 and T3.

*Proteobacteria* are often associated with inflammatory conditions [9]. Interestingly, with the findings of significantly higher levels of the proinflammatory cytokines IFN-g, IL-2, IL-6, and TNF-a in T3 than in T1, this would suggest that the T3 mucosal surfaces of the gastrointestinal tract present low-grade inflammation in advanced gestation [3].

Faecalibacterium, a butyrate producer with anti-inflammatory properties that is deficient in inflammatory bowel disease [10], and in patients with metabolic syndrome [11] is less prevalent in women with a normal pregnancy in the third trimester [3]. Even though the reduction of Faecalibacterium in this study was not statistically significant, it did show a reducing pattern.

This aberrant gut microbiota dysbiosis toward the third trimester of pregnancy was reported to be related to adiposity, low-grade inflammation, insulin resistance, and hyperglycemia independent of GDM status [3]. Its similarity with the changes associated with metabolic syndrome, however, seems to be a requirement of a normal healthy pregnancy.

Our study also did not find any significant difference at phylum level between women with GDM and NGDM; which similar to previously published studies [12, 13] *Bacteroidetes* was the dominant phyla in the GDM group throughout their pregnancy, and there is no predominantly Firmicutes trend as demonstrated in overall subjects or in NGDM group.

On the contrary, specific differences between GDM and normoglycemic women were reported by a few studies. The increased gut abundance of *Parabacteroides distasonis, Klebsiella variicola, Ruminococcus, Eubacterium, Prevotella, Collinsella, Rothia, Desulfovibrio,* Actinobacteria, Firmicutes and reduced gut richness of *Methanobrevibacter smithii, Alistipes* species, *Bifidobacterium* species, *Eubacterium* species, *Akkermansia, Bacteroides, Parabacteroides, Roseburia,* and *Dialister* were reported in GDM patients compared to normoglycemic controls [14].

However, at the genus level, apart from demonstrating *Bacteroides* and *Faecalibacterium* were the dominant genera representing more than 50% of the gut microbiota community structure in both GDM and NGDM groups, there was a discriminant pattern observed between GDM-associated and NGDM-associated gut microbiota (R^2^ = 0.59).

Among the 15 key genera with the highest VIP score (> 1.5) that contributed to the observed discriminant pattern of gut microbiota associated with gestational diabetes, *Acidaminococcus, Clostridium, Megasphaera* and *Allisonella* were found significantly higher, while *Barnesiella* and *Blautia* were found significantly lower in women with GDM. An elevation of bacterial genera from the class of Negativicutes such as *Acidaminococcus, Megasphaera* and *Allisonella* in GDM is also seen abundant in type 2 diabetes mellitus patients [15, 16]. The difference in gut microbiota could presumably be related to the metabolic changes during pregnancy, or perhaps, it could be due to distinct lifestyle and eastern dietary habits such as high carbohydrate and fat intake, and low fiber intake during the pregnancy. This suggestion was made as all the subjects had no known pre-pregnancy case of diabetes, metabolic syndrome or any other endocrine disorders. The findings may suggest the metabolic roles of these bacteria in adiposity, low-grade inflammation, insulin resistance, and hyperglycemia independent which required functional study confirmation.

Further analysis looking at the BMI of the participants in this study demonstrated a lower α- diversity among women who were obese, followed by pre-obese compared to women with normal BMI, even though this observation was not statistically significant. This was also observed by Koren O. et. al. (2012) who found that the women who were obese prior to pregnancy had the lowest within-subject α-diversity at both T1 and T3, although this was not significantly different from normal-weight women [3].

The gut microbiota's role in the pathogenesis of obesity has been clarified through studies in both humans and animal models [17]. However, no statistically significant differences in the relative abundances of each phylum between the BMI groups in this study. *Bacteroidetes* was found to be the most dominant phyla in obese (54.7%) and pre-obese (46.7%) groups. Bacteroidetes is a gram-negative bacteria, which is the largest contributor to lipopolysaccharides (LPS) production. As a result, increasing Bacteroidetes abundances during pregnancy may cause higher inflammation [18]. LPS can trigger inflammation through the Toll-like receptor 4 (TLR4) signalling pathway in preeclampsia [19], hence this could explain why obesity increases the risk of pre-eclampsia. This finding is in accordance with findings reported by Zhang et al. (2009) where they found there were more Bacteroidetes in the obese subjects than subjects with normal BMI 20]. This contradicts the earlier findings reported by Ley et. al. (2006) that obese people had lower Bacteroidetes and more Firmicutes than did lean control subjects [21]. Whereas Duncan et al. (2008) did not detect any differences between obese and non-obese individuals in terms of the proportion of Bacteroidetes measured in the fecal samples, and no significant changes in the percentage of Bacteroidetes occurred in feces from obese subjects even upon weight loss [22].

The generated PLSDA score plot, according to the BMI, showed a discriminant pattern of gut microbiota between two overlapping clusters. It demonstrated that in the obese group, *Megamonas, Succinatimonas* and *Dialister* were elevated whereas *Oscillibacter, Oscillospira, Butyricimonas, Alistipes,* and *Prevotella* were reduced. Two genera which are *Barnesiella* and *Blautia* were found reduced in both obese and pre-obese profiles. We could suggest that obese BMI gut microbiota during pregnancy is enriched with bacteria from a class of Negativicutes and Proteobacteria such as Megamonas, Succinatimonas and Dialister. An elevation of Negativicutes is also seen in the GDM profile from this study and observed in type 2 diabetes mellitus patients [15].

In a study of 81 stool samples from Taiwanese for analysis of the association between the gut flora and obesity, they found the most abundant genera of bacteria in cases with a BMI ≥ 27 were Bacteroides (29%), Prevotella (21%), Escherichia (7.4%), Megamonas (5.1%), and Phascolarctobacterium (3.8%) [23]. Similar dominance of Megamonas was demonstrated, however other bacterial dominance pattern was not the same. Megamonas also was found to be significantly higher in a study among obese children [24].

Whereas the normal and mild underweight BMI gut microbiota during pregnancy are enriched with bacteria from the class of Clostridia (*Papillibacter, Oscillibacter, Oscillospira, Blautia, Dorea)* and Bacteroidia (*Alistipes, Prevotella, Paraprevotella).* Some genus from Clostridia such as *Oscillospira* has been associated with leanness and low BMI as seen in 6376 participants from the Guangdong Gut Microbiome Project, China [25]. Several other animal and human studies also found a correlation between the high abundance of *Oscillospira* with lower BMI in lean mice and human subjects from several populations such as Colombia, USA, and Europe [26–30]. Nevertheless, our findings suggest there are potential metabolic links between Negativicutes, Clostridia and Proteobacteria with host parameters such as body weight which required further investigation.

### Limitation

This study has a few limitations that need to be considered. It has been reported that gut microbiota also influenced primarily by dietary intake [31, 32]. Lack of dietary information in this study, except for the absence of dietary modification by prebiotics, probiotics or antibiotics for 4 weeks as part of recruitment criteria, leads to an inability to correlate the gut microbiota profile with the dietary intake. This study also was not designed to match a disease and control, but rather an observational study to explore the gut microbiota profile among Malaysian pregnant women which has not yet been reported till date. Finally, the recruitment of the participant and faecal sample was done in both the first and trimester. Despite it covered the whole period of trimester which spanned three months, this was an acceptable method of data collection due to the variation of the gut microbiome within a trimester is negligible [3].

## Conclusion

There was no significant difference in gut microbiota composition between the first and third trimester among Malaysian pregnant women. However GDM and high BMI demonstrated significantly different gut microbiota composition at the genus level. Thus, our study was able to reveal the prevalence and variation of several key members of the gut microbiota and potential links between the dynamic changes in community profile with host parameters during pregnancy such as body weight and gestational diabetes status.

## Supplementary Information

**Additional file 1:** **Supplementary file 1.** Shannon rarefaction curve showed a plateau andsaturation phase.

**Additional file 2:** **Supplementary file 2.** PLSDA-VIP score (genus) list for GDM vs NGDM.

**Additional file 3:** **Supplementary file 3.** PLSDA-VIP score (genus) list for different BMIgroups.

**Additional file 4:** **Supplementary file 4.** Metadata of Bioproject and Biosamples of eachdatasets in public database NCBI.

## Data Availability

The datasets generated and/or analysed during the current study are available from the corresponding author upon reasonable request.

## Abbreviations

T1: First trimester
T3: Third trimester
GDM: Gestational diabetes mellitus
NGDM: Non Gestational diabetes mellitus
BMI: Body mass index
OGTT: Oral glucose tolerance test
WHO: World Health Organisation
