# Supplementary material for: Gut microbiota in pregnant Malaysian women: a comparison between trimesters, body mass index and gestational diabetes status
Source: BMC Pregnancy Childbirth. 2022 Feb 24;22:152. doi: 10.1186/s12884-022-04472-x (PMC8876553; doi:10.1186/s12884-022-04472-x)

Additional file 1: Supplementary file 1 Shannon rarefaction curve showed a plateau and saturation phase

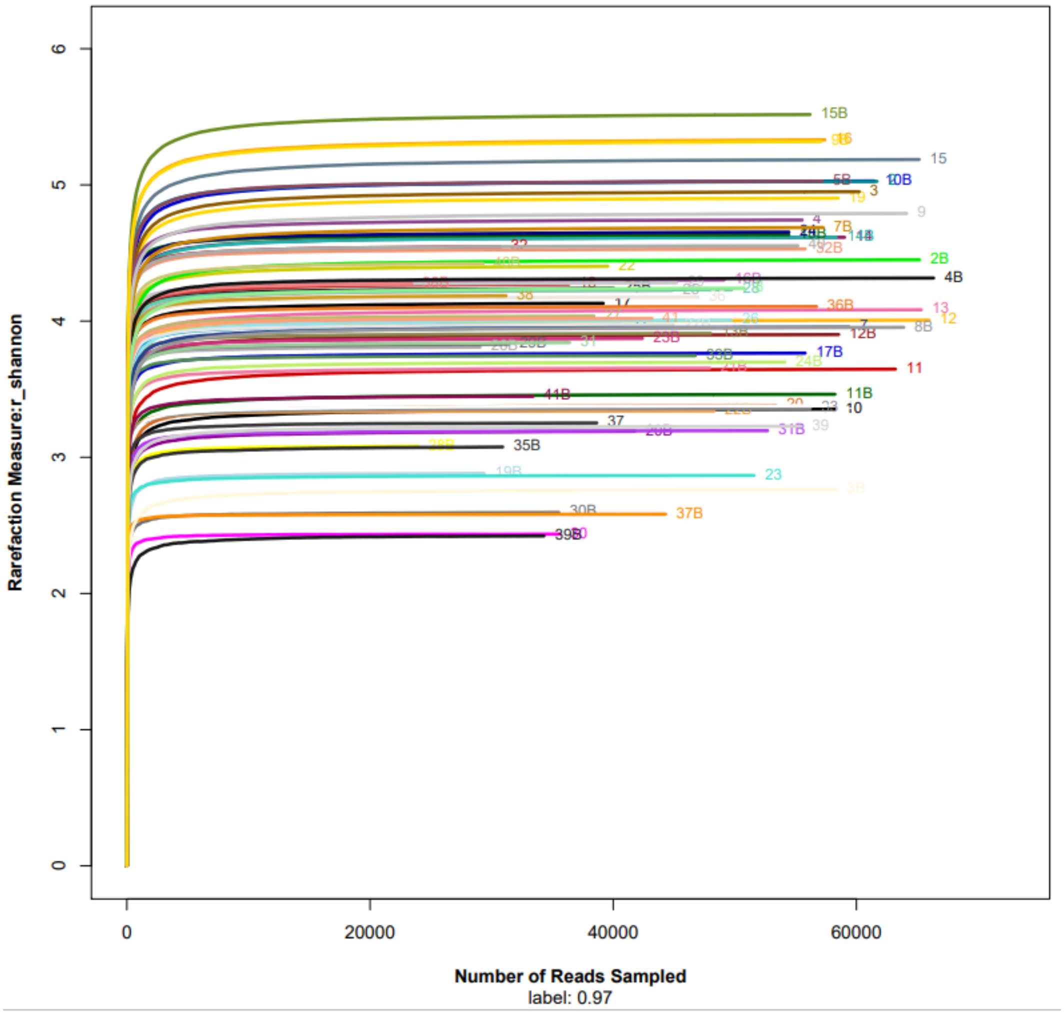

Supplement: Supplementary file 1 — Additional file 1: Supplementary file 1. Shannon rarefaction curve showed a plateau andsaturation phase. [file 12884_2022_4472_MOESM1_ESM.pdf]
